# Supplementary material for: Large-Scale Drug Screen Identifies FDA-Approved Drugs for Repurposing in Sickle-Cell Disease
Source: J Clin Med. 2020 Jul 17;9(7):2276. doi: 10.3390/jcm9072276 (PMC7408993; doi:10.3390/jcm9072276)
Supplement: Supplementary file 1 [file jcm-09-02276-s001.pdf]

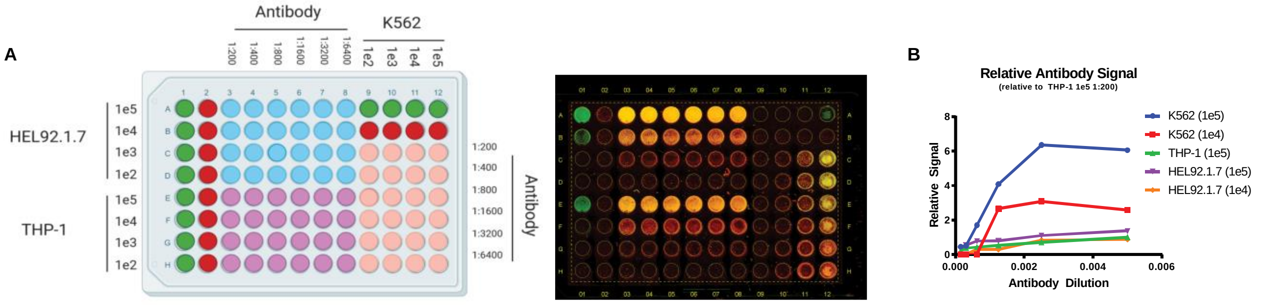

Figure S1. Condition validation of fetal hemoglobin antibody for in cell western. (a) Plate layout and sample fluorescent image generated. (b) Antibody quantification for fetal hemoglobin in differing cell concentrations for positive expression cell lines (K562, HLE92.1.7) normalized to negative expression cell line (THP-1).

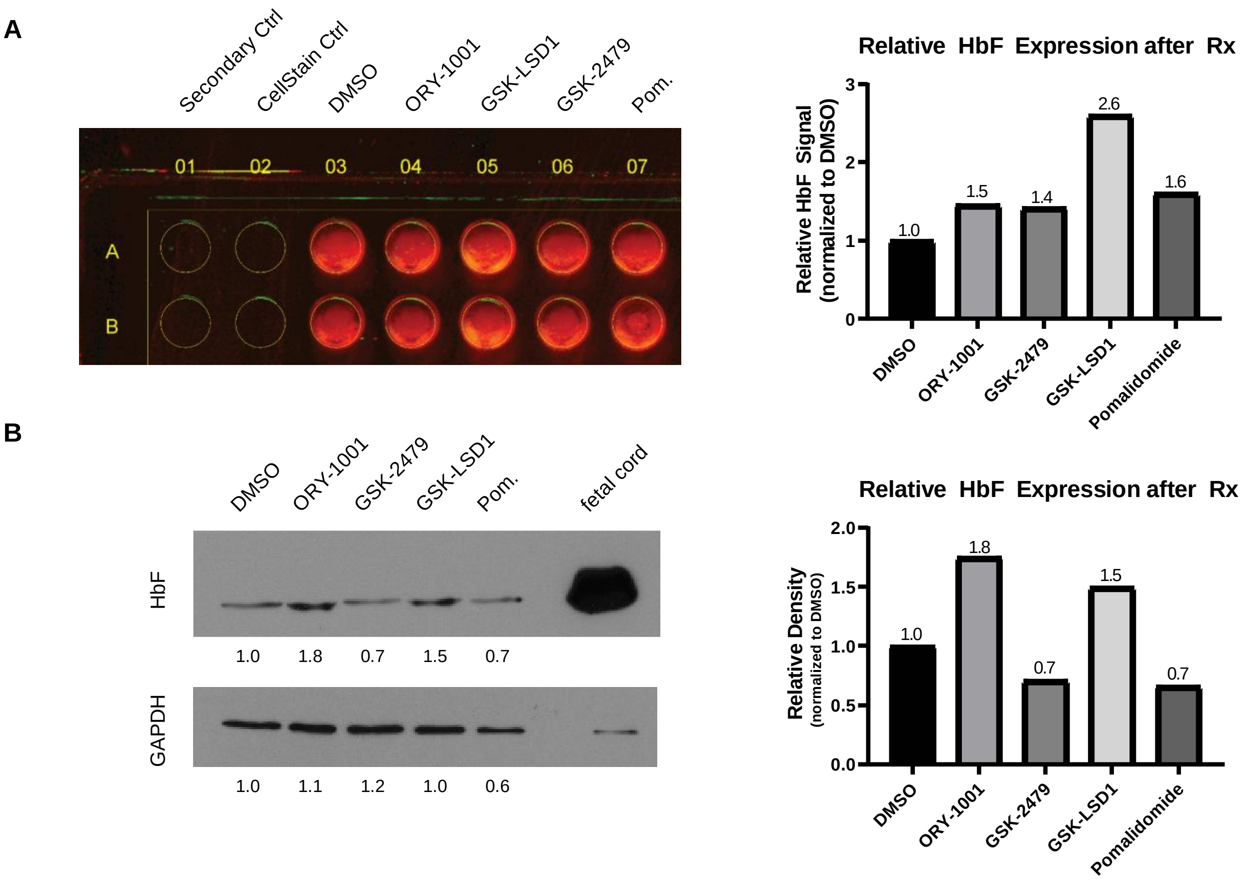

Figure S2. In-cell western correlates to traditional western with LSD1 inhibition. (a) In-cell western analysis and quantification of *in vitro* differentiated RBCs from sickle cell donor treated with LSD1 inhibitors. Quantification is normalized to DMSO. (b) Traditional western blot analysis and quantification of *in vitro* differentiated RBCs from sickle cell donor treated with LSD1 inhibitors. Quantification is normalized to DMSO.
